# Sensor array for wireless remote monitoring of carbon dioxide and methane near carbon sequestration and oil recovery sites

**DOI:** 10.1039/d0ra08593f

**Published:** 2021-02-10

**Authors:** Wesley T. Honeycutt, Taehwan Kim, M. Tyler Ley, Nicholas F. Materer

**Affiliations:** Oklahoma Biological Survey, University of Oklahoma 111 Chesapeake St. Norman OK 73019 USA; Centre for Infrastructure and Engineering and Safety, School of Civil and Environmental Engineering, The University of New South Wales Sydney NSW 2052 Australia; College of Engineering, Architecture and Technology, Oklahoma State University 201 ATRC Stillwater OK 74078 USA; Department of Chemistry, Oklahoma State University 107 Physical Sciences Stillwater OK 74078 USA nicholas.materer@okstate.edu +1-405-744-5920

## Abstract

Carbon sequestration and enhanced oil recovery are two important geochemical applications currently deployed using carbon dioxide (CO_2_), a prevalent greenhouse gas. Despite the push to find ways to use and store excess CO_2_, the development of a large-area monitoring system is lacking. For these applications, there is little literature reporting the development and testing of sensor systems capable of operating in remote areas without maintenance and having significantly low cost to allow their deployment across a large land area. This paper presents the design and validation of a low-cost solar-power distributed sensing architecture using a wireless mesh network integrated, at selective nodes, into a cellular network. This combination allows an “internet of things” approach in remote locations and the integration of a large number of sensor units to monitor CO_2_ and methane (CH_4_). This system will allow efficient large area monitoring of both rare catastrophic leaks along with the common micro-seepage of greenhouse gas near carbon sequestration and oil recovery sites. The deployment and testing of the sensor system was performed in an open field at Oklahoma State University. The two-tear network functionality and robustness were determined from a multi-year field study. The reliability of the system was benchmarked by correlating the measured temperature, pressure, and humidity measurement by the network of devices to existing weather data. The CO_2_ and CH_4_ gas concentration tracked their expected daily and seasonal cycles. This multi-year field study established that this system can operate in remote areas with minimal human interactions.

## Abstract 2

Demonstration of a solar-powered sensor array for remote carbon sequestration and enhanced oil recovery monitoring. An unattended sensor array can collect real-time gas concentrations, allow leak detection, and measure daily concentration cycles.

## Graphical abstract

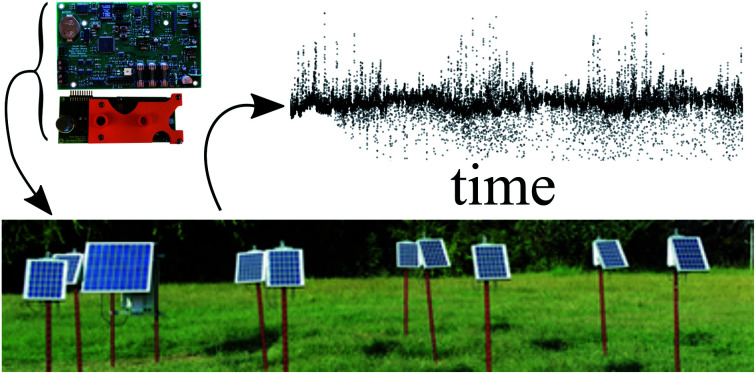

## 1 Introduction

The effect of carbon dioxide (CO_2_) in the atmosphere on terrestrial temperature has been known since the late 19^th^ century as quantified by Arrhenius.^1^ As CO_2_ output from anthropogenic sources increases,^2^ one scheme to combat atmospheric buildup is the underground injection of excess CO_2_ from significant waste streams, such as flue gas produced by coal-fired power plants. Injected gas can be stored in naturally occurring subterranean voids, or those left by mining and hydrocarbon production sites.^3,4^ Studies suggest that carbon sequestration by injection has a net benefit in terms of greenhouse gas (GHG) mediation efforts.^5^ The injection of pressurized CO_2_ gas has also been found to liberate hydrocarbons, enabling enhanced oil recovery (EOR) from new or existing wells. Once injected, CO_2_ liberates valuable hydrocarbons including methane (CH_4_).^6^ A report from the National Energy Technology Laboratory of the United States Department of Energy claims the sequestration of 20 billion metric tons of CO_2_ has been employed as part of EOR programs since their discovery.^7,8^ As of 2014, 53% of all commercial-scale EOR projects in the United States utilize gas injection techniques,^9^ and the number of projects using this method is expected to increase as there is more push for CO_2_ sequestration in conjunction with rising energy prices.^10^

There are environmental, health, and safety concerns regarding the potential outcome of injected CO_2_.^11^ In a process known as microseepage,^12–14^ small quantities of CO_2_ can slowly seep out of the reserve. Understanding this seepage is an important consideration for determining the economic feasibility and the development of potential regulation for injection sites.^15^ In addition to microseepage, a large leak of the sequestered CO_2_ can potentially result in a catastrophic event with costly consequences. Displacing oxygen, a cloud of CO_2_ has the potential of asphyxiating all animal life in an area, as evidenced by the events at Lake Nyos^16^ and Lake Monoun^17^ in Cameroon which caused massive loss of human life. For EOR, the monitoring of CH_4_ is critical since it can leak in concert with CO_2_. Biogenic reduction of CO_2_ to CH_4_ has led to seepage of CH_4_ from non-EOR injection sites.^18^ As CH_4_ has a greenhouse impact 84 times greater than CO_2_,^19^ understanding CH_4_ leakage from injection sites is also important from an environmental health perspective.

To date, researchers have relied heavily on computer modeling to address risks, to understand microseepage, and to estimate the environmental impacts. These models have been shown to be effective for predicting the lateral diffusion underground^20,21^ through discrete geologic layers, assuming only minimal vertical migration.^22^ Since studies have found that tracer molecules injected at one site can be detected at neighboring drill sites with time, such assumptions made in the modeling should be reexamined to ensure that the computational result match experimental measurements.^23,24^

To address both environmental and health and safety concerns, comprehensive monitoring of both CO_2_ and CH_4_ at the injection site and the surroundings area is needed to quantify the long term impacts. Monitoring stations in development for the In Sallah injection well and have been shown to detect simulated leaks, but these sensors are only capable of single site monitoring as of this publication.^25,26^ Recently, unmanned aerial systems have been of particular interest for remote monitoring of large areas.^27^ However, these solutions are not capable of continual monitoring over longer time scales and the aircraft require continuous maintenance from trained personal. Hyperspectral imaging, light detection and ranging (LIDAR), and other similar technologies working in combination can also be used to detect leaks.^28,29^ A study by NASA of a CH_4_ leak has shown the modern LIDAR equipment was even able to measure an above-ground gas plume from an exceptionally large leak.^30^ Nevertheless, the authors found no published studies of CO_2_ or CH_4_ leakage from carbon sequestration sites over long time scales due, in part, to the lack of low-cost instrumentation that can efficiently be deployed to provide local coverage around physically remote area. This paper presents the design, implementation, and deployment of a sensing array for this application.

## 2 Sensing array implementation

The sensing array's design aim is to provide dense local coverage of CO_2_ and CH_4_ around physically remote areas of interest, such as CO_2_ injection sites. The sensor network also follows a “lightweight” design paradigm to allow the array to be deployed and rearranged with minimal effort. These constraints are distinguishable by this implementation from those used for other environmental monitoring stations, such as those used to monitor weather (Mesonet) or Chemical Speciation Network (CSN) each with significant infrastructure investment at fixed locations.^31–33^ With this philosophy in mind, a low-cost solar-powered distributed sensing architecture using a wireless mesh network integrated, at selected nodes, into a cellular network, was designed, and implemented at the test site. The resulting array was validated by continuous operation since 2015.

This effort builds on wireless sensor array concepts used in agricultural applications to monitor local conditions^34^ and aerial cloud analysis.^35^ The implemented sensor array is organized into three tiers. An overview of the tiered hierarchy is shown in Table 1. Tier 2 sensors are self-powered and portable (smaller than a backpack and weighing less than two typical textbooks) and, upon activation, self-assembles a mesh network based on the sub-network the device was assigned during activation. Assignment to sub-networks allows for multiple local networks to be implemented within overlapping radio ranges. In this paper, a DigiMesh (Digi International, Hopkins, MN) protocol coordinated the mesh networking, but the work described here is generally applicable to any network implementation capable of creating an addressable local-area network with low-power digital radios (*e.g.* Zigbee). Each of these local networks are limited to approximately 50 devices per network to ensure network reliability, with a unique identification number. Every mesh network, principally comprised of Tier 2 nodes, contains a single, larger Tier 1 node. Tier 1 nodes are similarly self-powered devices with additional sensors and cellular connectivity, requiring a larger solar panel and battery capacity to meet the power consumption requirements. Due to these additions, they are less portable than the small Tier 2 devices. This larger node coordinates with the sensors in its local network to transmit measurements over the cellular data network. Importantly, this scheme allows addition of sensor elements with high power consumption to operate in conjunction with the low-power sensors on the Tier 2 nodes. In the presented implementation, the Tier 1 nodes have extended sensing capabilities for CH_4_. The highest level of network architecture, Tier 0, is an internet server, a workstation housed at the base of operations, and this device is connected to a municipal power supply and the internet. The Tier 0 server acts as a receiver of the field data and has the computational ability to process and analyze the data. This tiered node architecture allows for a large number of inexpensive solar-powered sensors to monitor CO_2_ and CH_4_ with maximal area coverage.

**Table 1:** Hierarchy and respective requirements of devices in the network array designed for this project

Tier and name	Device requirements
Tier 0 – internet server	Large storage space
Tier 1 – communication node	Simple gas sensors
	Advanced gas sensors
	Moderate power supply
	Wireless communication
	Cellular modem
Tier 2 – sensor node	Simple gas sensors
	Low power supply
	Wireless communication

### 2.1 Hardware overview

**Fig. 1:**
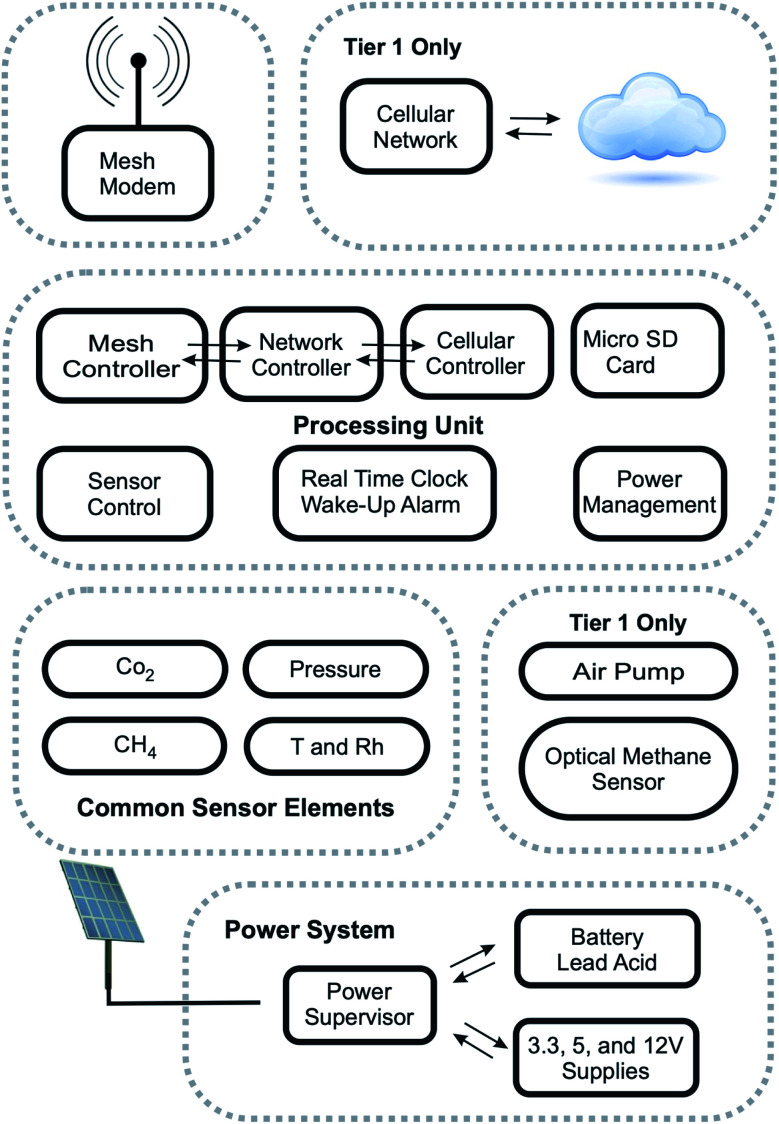
A generalized representation of the functional blocks within the Tier 1 and 2 nodes. The blocks are: the communication sections (“Mesh Modem” and “Cellular Network”), the “Processing Unit” with local storage, sensors (“Common Sensor Elements” apply to Tier 1 and 2 units, but functional group containing the “Optical Methane Sensor” and its air pump only apply to Tier 1 units), and the “Power System”.

The Tier 1 and 2 nodes can be generalized by a set of functional blocks, such as depicted in Fig. 1. By design, the Tier 1 and 2 nodes share a significant amount of functionality at the hardware level, including the same control board containing the mesh communication modem, the local storage, and power management circuitry. For the Tier 1 node, a Skywire EVDO cellular modem (Nimbelink, Plymouth, MN) was mounted on a separate breakout board, connected by a ribbon cable, to the same control board used by the Tier 2 nodes. In addition to the cellular connectivity, the Tier 1 node contains an additional sensor functional unit to allow active sampling using an optical methane sensor. For a small production of approximately 100 Tier 2 and 10 Tier 1 nodes, the unit costs are estimated at $550 and $3500, respectively. The Appendix include a detailed cost estimate (Table 2), images of the device enclosures (Fig. 10), control board (Fig. 11), the common sensor board (Fig. 12), and the sensors deployed at the field site (Fig. 14). The complete electronics schematics described here, as well as an in-depth discussion of each component, are publicly available in the Honeycutt dissertation.^36^

### 2.2 Common sensor elements

For low-power and low-cost environmental sensors, the specific needs of the collected data must be weighed against the complexity and cost of the integrated system. The Tier 1 and Tier 2 nodes share a set of the inexpensive sensors mounted on a common sensor board (“Common Sensor Elements” in Fig. 1). To minimize power expenditure, the Tier 2 nodes passively sample the environmental conditions.

Thus, the sensor board is directly exposed to the environment. To protect against potential issues, including moisture, animal life, and physical impacts, the exposed sensor board is mounted on a 3D printed plastic housing, shown in Fig. 13 in the Appendix. Since the Tier 1 sensors include an additional sensor which requires pumped air, the sensor board is enclosed within an airtight box with adequate volume to allow for passive sampling of the same gas is actively pumped from outside (see Section 2.3).

The common sensor board includes elements to obtain an environmental baseline for performance validation in addition to the low-cost CO_2_ and CH_4_ sensors. These include an SHT75 temperature and humidity sensor (Sensirion AG, Zurich, Switzerland) and a MPXA6115A pressure sensor (NXP Semiconductors, Eindhoven, The Netherlands). For the CO_2_ and CH_4_ sensors, Honeycutt *et al.* reports limit of detection and precision of various low-cost commercially available sensors.^37^ Based on these results, the common sensor board includes a K-30 sensor (CO2Meter Inc., Ormond Beach, FL) for CO_2_ and an MQ-4 sensor (Futurelec, New York, NY) for CH_4_. The K-30 sensor was picked for our system due to performance, low-power, and low-cost. In the case of CH_4_, there is no commercially available sensor that provides parts-per-million sensitivity at atmospheric levels, with low power consumption, low price, and easy availability. Previous work by Honeycutt *et al.*^37^ compared several solid-state CH_4_ sensors, including those from MQ-4 from Hanwei Electronics and TGS-2600, TGS-2610, and TGS-2611 manufactured by Figaro Engineering Inc. These sensors are not low-power, due to the presence of a heating coil, and require power management. The detection limit of the MQ-4, TGS-2600 and TGS-2610 were similar. Previous measurements for the TGS-2611 sensor show that it was slightly superior by about a factor of 5 to 10 with respect to its detection limit when compared to the other sensors.^37^ The MQ-4 was chosen due to its availability in larger quantities (100 s) within mandated the time period to construct the array and perform this study. The selected sensor are sufficient to detect potential leaks (defined as 1000 ppm and above) from carbon sequestration and EOR sites.

### 2.3 Tier 1 sensor elements

The higher power storage capacity means the Tier 1 node can include additional sensors for better performance. Given a previous study,^37^ a Gascard CH_4_ sensor (Edinburgh Instruments Ltd., Livingston, UK) was chosen for inclusion in these nodes. The Gascard CH_4_ sensor requires a mechanical pump for sampling, which can be turned off to save power when required. A long-life 1410D/2.2/E/BLDC diaphragm pump (Gardner Denver Thomas GmbH., Munich, Germany) was used to pull air from the outside of the enclosure through a hole on the underside. A 0.45 μm particle filter protected the pump and the sensors from particulate matter. The air is passed through the Gascard CH_4_ sensor, then to an acrylic enclosure containing the common sensor board, before being finally exhausted outside the enclosure. The air inlet and outlet ports are placed on opposite ends of the bottom side of the enclosure to minimize recycling of analyzed gas.

### 2.4 Sensor calibration

The details of the calibration and baseline analysis is reported in ref. 37. Briefly, all gas concentration sensors used in this study were calibrated in a laboratory setting prior to deployment. Sensors, placed in a mixing chamber, were subjected to varied mix ratios of calibration standard (certified within ±2% by Airgas Inc.) and a carrier gas, either nitrogen or clean, dry air. The sensors were calibrated from 0 ppm to a maximum of 3000 ppm of the relevant gas. The sensor response was compared against a high sensitivity ZRE Non-Dispersive Infrared Analyzer from California Analytical Instruments, Inc. Optical sensors were found to have a linear calibration curve, while chemiresistive sensors were found to have the expected non-linear calibration curve.

### 2.5 Control board

The control board contains an Atmel ATMega2560 (Atmel Microchip, San Jose, CA) low power 8 bit microcontroller with 64 kB flash operating on a RISC architecture (referred to as ATMega 2560 in this manuscript). The ATMega 2560 is under-clocked to save power using an MA-506 (Seiko Epson Corporation, Suwa, and Nagano Prefecture, Japan) with an 8 MHz ± oscillator. The software was written in the Arduino environment with additional low-level code to allow the processor to turn its oscillator off and to enter a deep sleep mode when not in use. To realize this savings all functions are interrupt driven. A DS3231 real-time clock (Maxim Integrated, San Jose, CA) and coin cell battery is used to create logging timestamps and to generate an interrupt to wake the processor every second. The DS3231 also provides an accurate clock, critical when data are being transferred across the network from multiple sensors. The Tier 0 server is responsible for ensuring that all clocks are set correctly. Data are logged using two memory storage methods. The first is a 24LC1026 1024 kB serial EEPROM (Microchip Technology, Chandler, AZ). The second is a micro Secure Digital (SD) stable storage card, placed in a SCHD3A0100 micro SD card holder (Alps Electric, Ota, Tokyo, Japan). The SD card can be disconnected from the power supply when not writing or reading data to reduce energy consumption.

For node-to-node communication, the board contains a socket for a XBee-Pro 900 HP radio (Digi International, Hopkins, MN). A serial interrupt to wakes the processor when a data packet is available. The board also provides a serial interface and power management for the cellular modem and the Gascard sensor used in the Tier 1 node. A 34-pin ribbon cable interface is also provided for the common sensor board described above. The cable provides a digital serial communication channels for the K-30 sensor and SHT75 sensor. The cable also connects the pressure and MQ-4 sensors to an analog filter, then to a dedicated 12 bit analog to digital converter on the control board. Both the K-30 sensor for CO_2_ and an MQ-4 sensor for CH_4_ can be independently turned off to save power.

### 2.6 Tiered communication scheme

The Tier 1 and 2 devices are designed to allow dense local coverage of CO_2_ and CH_4_ sensors in a remote area with minimal setup. The array must be deployable and rearrangeable with minimal effort and skill. Thus, a modem network capable of self-assembly was chosen. In the mesh network, data packets can transfer across multiple Tier 2 nodes as they travel to the Tier 1 node, increasing the range and allowing easily deployment without worrying about network constraints. The raw data format is an UTF-8 encoded hexadecimal string generated from the binary data from each network element. The stream also includes routing information (device addresses and network identification), timestamps, power status and current draw of each individual sensor element, solar panel and battery status, data payload, network status, and CRC16 checksum. Adhering to best-practices, the checksum records origin, transport nodes and integrity of each packet. Both the Tier 1 and 2 communication implementation uses the packet acknowledgment feature of the mesh network to ensure reliable transmission. The network status code includes these acknowledgments along with a flag indicating that it been written to an SD card. The automatic network routing allows any Tier 2 node to route packets originating from other Tier 2 nodes which are too far to allow these packets to be directly received by a given Tier 1 node.

As a means of congestion avoidance, each Tier 2 sends data packets at predetermined minute-long windows within a given hour. The network has a large latency (on the order of seconds) following the first packet sent in each transmission. However, the next packets are sent quickly. The CPU communicates with the modem using a 9600 BAUD serial link. Transmission between the digital modems occurs at a maximum rate of 200 kbit s^−1^. Thus, even in less than ideal conditions, it will never reasonably take longer than a minute to complete the transmission, even if this is the first successful data transmission event in a given month. Delays longer than a month are unlikely. Such delays would imply highly overcast skies for the same period or damage to the equipment which would be indicated by a lack of packets for a particular node received by the Tier 0 node. In areas where large delays are possible, additional functionality to prevent transition and require directly accessing the local SD card, which is possible from Tier 0 node, should be implemented. In the current setup, minutes :01 through :50 are reserved for sending data packets while minutes :51 to :60 are reserved for Tier 1 to issue commands to Tier 2 devices. Thus, the theoretical limit for the communication scheme described is a maximum of 50 Tier 2 units assigned to a single Tier 1 unit. The authors have confirmed operation networks containing 30 Tier 2 nodes empirically. During the final ten minutes of the hour, commands can be issued by the Tier 0 server to the Tier 1 node, which may be further disseminated a selected Tier 2 node. Critical commands for network functioning include time synchronization and transmission time allotment. Additional commands include sensor parameter adjustments, power management parameters, and direct access to data stored on any local SD card. The reserved time ensures that any command can be completed before the data packets start being sent over the network again. This time partitioning proved to be very reliable over several years of continuous operation.

**Fig. 2:**
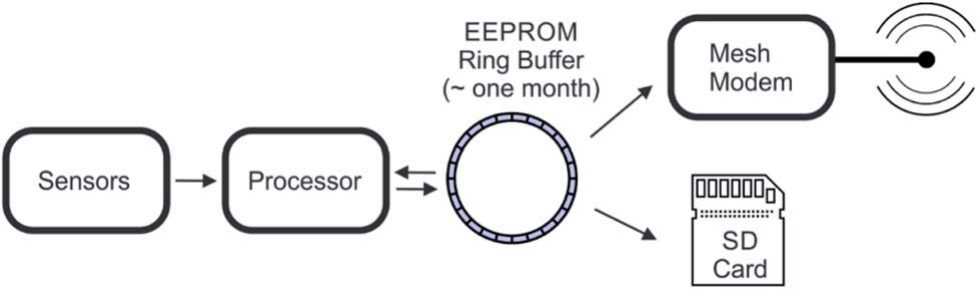
Block diagram of the data collection process on a Tier 2 sensor node.

In the Tier 2 units, the unprocessed sensor data and diagnostic information are collected every 15 min and stored in a ring buffer using the EEPROM component of the main circuit (see block diagram in Fig. 2) before been written to the SD card and transmitted to the Tier 1 node. Increasing the data sampling rates in situations of high target gas concentration has been considered but not implemented. Data in the ring buffer are sent to the Tier 1 node during the assigned transmission window. Once sent, a flag is set on each measurement to indicate successful transmission, as indicated by an acknowledgment packet from the Tier 1 node. The flagging allows manual data retrieval or future network reads to be easily merged with the successfully transmitted data. *In lieu* of automatic repeat requests for data which are not acknowledged, devices send unacknowledged data again on during the next transmission window. The final data packet also contains the collection time, the transmission time, and the time received by the Tier 0 node. This accounting proves critical when debugging the software and when reconstructing the data log from packets delayed due to power management events or communication errors during live deployment. Transmission of each data packet consumes significant power, and reduced power reserves can reduce transmission range, resulting in dropped packets. In these low-power situations, the ring buffer allows the system to delay transmission to the Tier 2 node until power reserved are replenished. The power-managed SD card provides a local backup and allows a record to be stored even in during extended times of cloudy weather. Depending on the battery charge, data sent to the Tier 1 node is transferred from ring buffer to the SD card once an hour (see block diagram in Fig. 2). As SD cards can use up to 100 mA during writes, the system can wait until the next specified write time if the power reserves are low. The risk of data loss is minimized given that up to one month of data can be stored in EEPROM before the data must be transmitted and written to the SD card. A file rotation is performed weekly to ensure that individual files on the SD card are kept at a reasonable size.

**Fig. 3:**
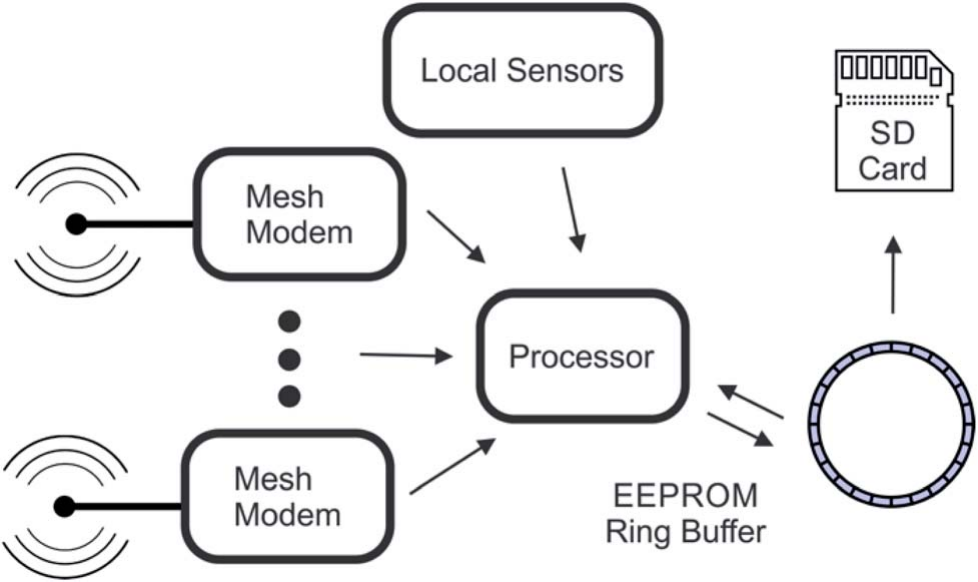
Block diagram of the data collection process on a Tier 1 communication node.

In the Tier 1 units, the unprocessed sensor data and diagnostic information are also collected every 15 min and stored in a ring buffer. In addition, the Tier 1 node also listens for data packets being sent over the mesh network. Each packet is acknowledged and stored in a ring buffer. Once in this buffer, the process of writing to the SD cards is identical to the Tier 2 node. Power management is also handled similarly, however the large energy storage capacity of the Tier 1 nodes ensures that it will not enter a power management mode before the Tier 2 nodes under identical solar flux conditions. The block diagram in Fig. 3 summarizes this process.

**Fig. 4:**
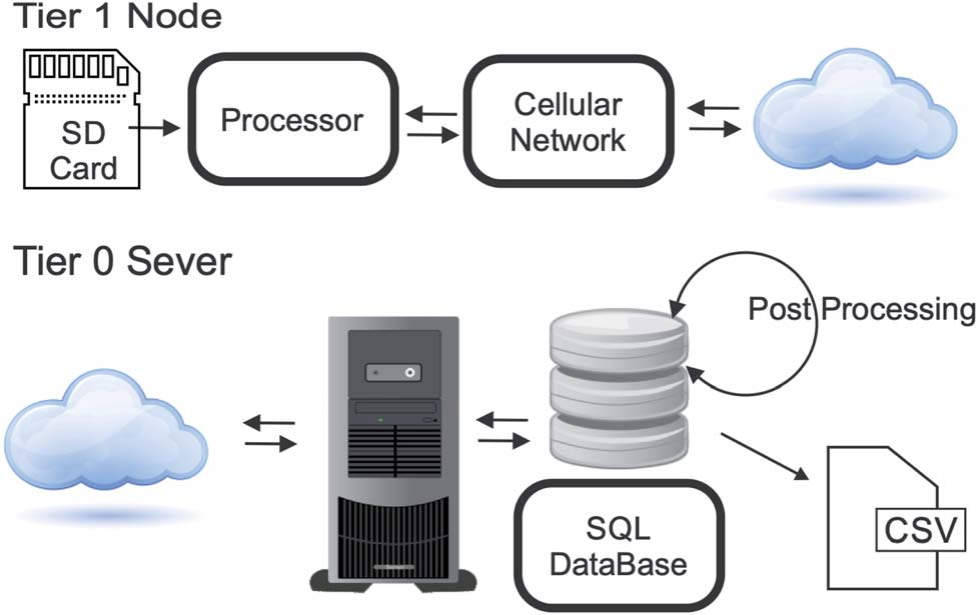
Communication to the Tier 0 server from each Tier 1 node.

The Tier 1 nodes transmit the directory of the SD card containing the collected data from all Tier 2 nodes, to the server (Fig. 4) once per hour *via* connection to the cellular data network. Given the large bandwidth of the cellular network, band congestion is not a problem, so each Tier 1 node connections can overlap in a given time window. Once connected, the node sends its unique mesh modem's 64 bit identification number and its current internet address to the Tier 0 node. The Tier 0 node uses the identification number to compare file sizes of data received during previous transmissions and requests any new data based on file size increase. As the data are stored sequentially in the data files, a file size increase is entirely due to additional lines of data. The final line count is recorded by the server along with the new file size to ensure that the next data transfer continues where the current transfer left off. Alternatively, for debugging purposes or to recollect data, the complete files can be requested from the Tier 1 node or, through the Tier 1 node, any Tier 2 node through the mesh network by means of special commands. Once the data are collected, queued commands for sensor management are sent to the Tier 1 node for distribution to Tier 2 nodes.

The Tier 0 server stores the raw data from the binary streams in a SQL database. The validity of each data packet is conformed through a simple CRC16 checksum generated during data collection. This second step allows all checksum to be validated, and a variety of post-processing to be performed without modifying the raw data. In addition, sensor configuration data (*i.e.* sensor location, power management parameters) and network parameters are also stored in a SQL database. These “vitals” data may be accessed separately from the collected raw sensor data. This feature is useful for remote monitoring of the sensor network through an internet portal. Periodically, the Tier 0 server processes the raw data to obtain the measured values from the sensors and other monitoring points. For further data analysis, this information may be downloaded from the server as a plaintext comma delineated data file.

### 2.7 Power system

The implementation aims at using a network of solar-powered sensors to monitor CO_2_ and CH_4_ in remote areas. Thus, each unit must have self-sustaining solar-powered supplies that can function for 5 years or longer. Weather data statistics show that 32% of the year is at least half-cloudy and 8% of the year is heavily overcast at the selected field site.^38^ Thus, it is possible to have times where not enough power is available to run all the available sensors, and power management must be an integral part of the sensor elements and the network implementation. Some aspects of this management have already been discussed. To this end, different solar power generators, power storage units, and weatherproof enclosures for both Tier 1 and 2 sensors were selected from Tycon Power Systems (Bluffdale, UT) (see Fig. 10 in the Appendix) Each enclosure has a battery, solar panel, voltage regulator, and charger system, which is programmed to shut down in low-power situations created by insufficient sunlight to maintain a charge on the internal battery. However, a hard shutdown is intended to protect the enclosure's battery, not the Tier 1 and 2 electronics. Another complication is that the hard shutdown sometimes cannot be reversed upon device recharge without physical user intervention.

In this implementation, the power management system on the control board avoids a hard shutdown due to low battery conditions by selectively deactivating individual energy-expensive sensors based on the battery's change. A “sleep mode” is automatically triggered by the control program if the battery is below 70% charge. In sleep mode, the device still uses power to regularly check in with the communication network. The final mode is a “hibernation mode,” with and prevents the units from requiring manned intervention during long periods of overcast conditions. It is activated by very low battery charge, as indicated by a threshold voltage. Once the battery voltage dips below this threshold, the unit deactivates nearly all components and timed events to prevent complete power drain. This mode pauses the communication check in and deactivates the sensing elements. Only 0.1 mW are used to periodically monitor the battery voltage in order to decide if it can exits hibernation mode.

## 3 Results

A small network of sensors was deployed on the Oklahoma State University campus (see Stillwater, OK deployment in the Appendix) to evaluate the device network. These sensors have been actively returning data since October 20^th^ 2015.

**Fig. 5:**
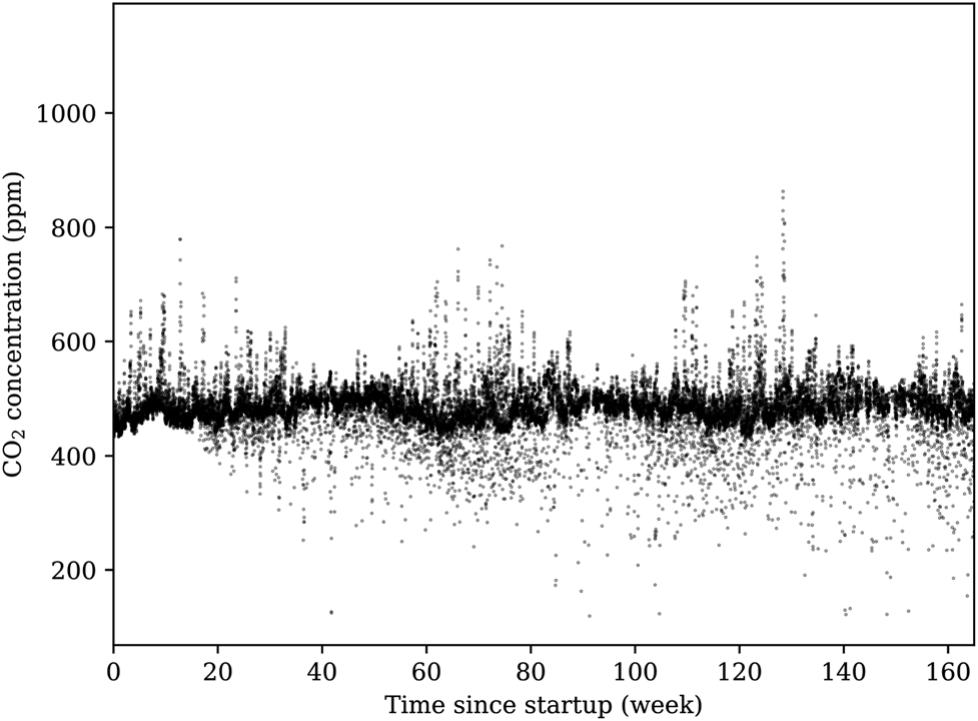
This figure shows the averaged results of the sensor network, per hour, after more than 3 years of continual operation. While the majority of measurements center on a site average, there are notable deviations both above and below this region. All reporting units in the network agree, within reasonable tolerance, on these deviations from the average site measurement. Close inspection of the results shown in this figure reveals several gaps in reporting, which indicates that units were powered down for a period before automatically restarting when power reserves reached the critical threshold.

The results reported in Fig. 5 demonstrate the reliable reporting of the unmanned sensor network over a long time period. There was no averaging or smoothing of the raw data. Similar figures can be generated using the pressure, temperature, relative humidity and methane data. These results will be discussed in the next section.

Over this time period, all sensors in the array operated reliably after brief periods of inactivity during low-power conditions and after changing weather conditions. During cloudy conditions, heavy rain, and winter and summer temperature extremes, all units continued to function properly. In one severe weather event, some of the units were even struck by flying debris from wind gusts exceeding 110 km h^−1^, and the enclosures suffered heavy cosmetic damage. Even during these extreme conditions, the array stayed operational. The long term data also shows significant fluctuation around the average valve, with more fluctuation toward lower CO_2_ concentrations. These fluctuations could be do to animal movements (*i.e.* deer), sensors powering down and then up due to solar power limitations, and potential effects of temperature, pressure and relative humidity on the reading of the sensor. Large fluctuations from the average are also possible during conditions of high relative humidity or under rapidly changing weather conditions. See Section 4.2 for more discussion. Importantly, all fluctuations from the average are under 1000 ppm, a level expected from a significant leak from a carbon sequestration or EOR site. Given this application, the described sensor design performed exceptionally well.

## 4 Discussion

Carbon sequestration and EOR are two important geochemical applications for remote sensor systems capable of operating in remote areas without maintenance and having significantly low cost. The selected sensors are sufficiently sensitive to detect potential leaks (defined as 1000 ppm and above) from carbon sequestration and EOR sites. In this discussion, the collected atmospheric data are compared to both expectations and data collected at local weather stations. The data reported by the sensor network is considered “good” if those reported by each sensor tracked well with the other sensors in the network, relying on previous studies of accuracy and precision for sensor quality assumptions.^37^

### 4.1 Temperature, pressure, and humidity

**Fig. 6:**
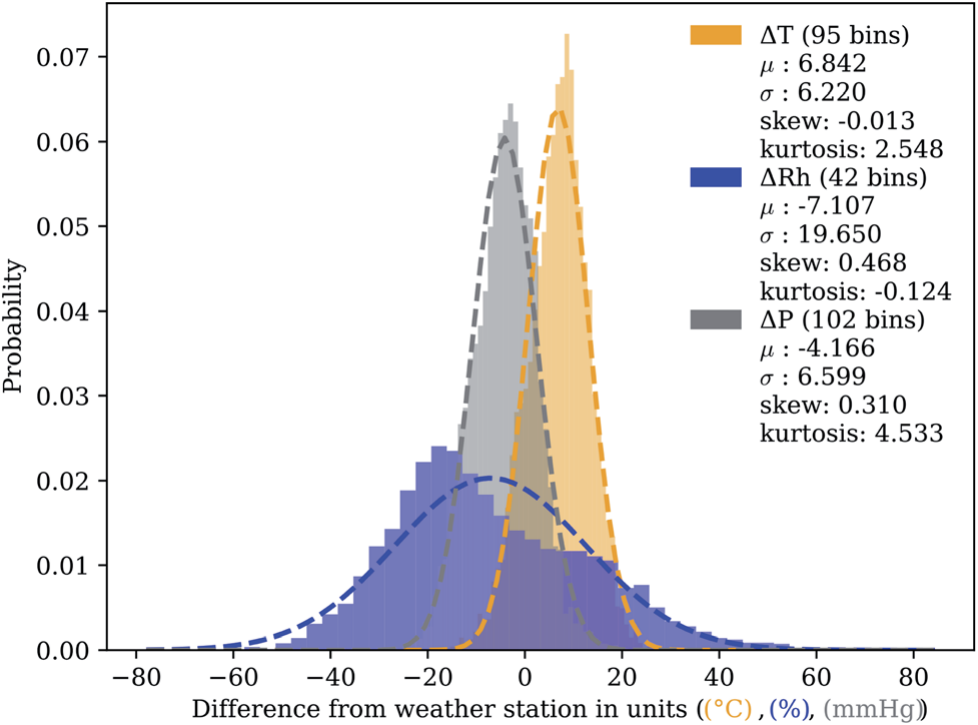
Temperature, relative humidity, and atmospheric pressure data from networked sensors was compared with data archived from KSWO.^39^ The plots represent the distribution of residuals between the reported KSWO hourly average and the total sensor network hourly average. Each plot includes information about the number of bins, determined by Freedman–Diaconis rule,^40^ plus values for relevant statistical descriptions of a fit including mean (*μ*), standard deviation (*σ*), skew, and kurtosis.

Data collected from the temperature, pressure, and relative humidity sensors were evaluated with respect to known weather data collected from the database of the U.S. Climate Reference Network's (USCRN) local climate data for Stillwater, OK.^39^ The closest weather station in the USCRN, Stillwater Regional Airport (KSWO) (36.1624°N, −97.0894°W, 299.9 m), reports hourly data, and an additional nearby station (Stillwater 2 West (36.1181°N, −97.0914°W, 271.3 m)) reports data every five minutes. To compare the sensor network collectively with the weather data, temperature, pressure, and relative humidity were averaged across all units and for all points within an hour. By taking the difference of these hourly points from the hourly average reported by KSWO, we generate residuals to compare the two. Fig. 6 depicts the distribution of residuals around the mean, bin sizes determined by the Freedman–Diaconis method, with relevant statistical results of a Gaussian fit of each distribution.^40^ Repeating the distribution analysis with the 5 min data from the Stillwater 2 W station showed no appreciable difference from the hourly sampling from KSWO. The peak of each distribution is shifted slightly from the mean value reported, and the difference is likely due to the difference in site elevation. While the surface of the sensor site stands at 287.7 m above sea-level, the KSWO instrumentation sits 299.9 m above sea level. The inverse shift of pressure and temperature peaks supports this argument.^41^ Although relatively simple to correct pressure for altitude, correcting temperature for altitude is not possible.

**Fig. 7:**
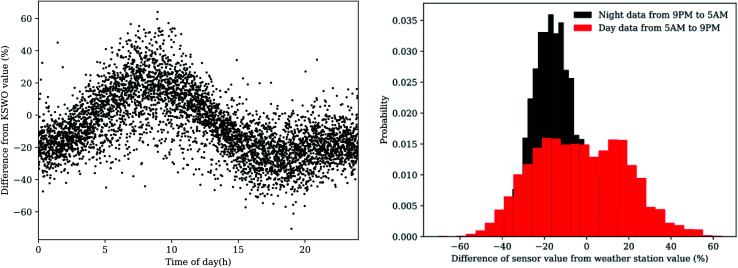
Humidity variation from KSWO occurs more during daylight hours.

The apparent bimodal distribution of humidity residuals at the site suggests altitude differences do not account for everything. Controlled environment tests, the SHT75 sensors faithfully recorded humidity within the bounds (±1.8%) stipulated by the manufacturer. By correcting the humidity residuals for time of day, we can plainly see in Fig. 7a a diel pattern appear. A daily cycle in the variation of humidity between two sites suggests that the local environment at the OSU testing site may naturally exhibit fluctuating humidity compared to the KSWO weather station. If we split the data into day and night portions (from 5 AM to 9 PM and *vice versa*), we see that the distribution from KSWO, depicted in Fig. 7b, neatly splits into separate normal distributions. The discrepancy in reported relative humidity between the sensor network described in this paper and KSWO appears to depend on the sun to alter local environmental conditions. There are many other factors to explain this variation, including prevailing wind direction, hydrologic features (a small creek and a pond near the test site), anthropogenic presence (including a nearby large petroleum fractionation tower), sensor location (near plant matter at this test site and on a tall tower at the airport), and other factors not explored in detail here.

### 4.2 CO_2_ concentration

**Fig. 8:**
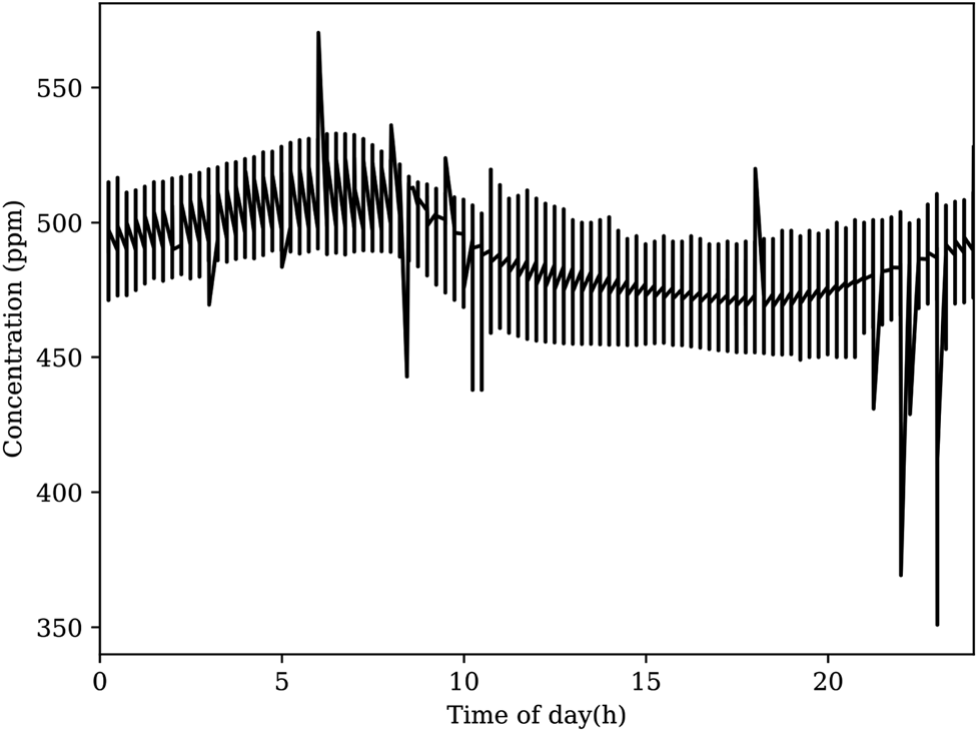
The average CO_2_ concentration reported by the sensor network at defined time points produces the expected diel cycle. This plot appears to show notable peaks and troughs outside the average curve, however these likely do not represent realistic events. Fourier analysis suggests that these spikes do not appear consistently, and they likely originate from outliers in the original dataset.

There is no local reporting agency for CO_2_ concentrations, thus validation of the concentration values reported by the sensors is not as simple to perform. As there are no other atmospheric gas sensors deployed in the atmospheric layer and geographic locale of our sensor network, we must approach conclusions in an indirect manner by verifying sensors with known equivalents nearby and considering sensor limitations. To help with the analysis, averaging the time points for the whole data set (see Fig. 5) yielded concentration plots as a function of time of day for CO_2_ shown in Fig. 8. This figure reports the average daily cycle of the analyte gas in the region.

For CO_2_, the global baseline value recently passed 400 ppm, yet the daily cycle of CO_2_ at the OSU campus exceeds 500 ppm most days near sunrise.^42,43^ The K-30 CO_2_ sensors are delivered with factory calibration, using built-in calibration and zeroing functions. These sensors also contain a membrane between the optical chamber to prevent particles and water from effecting the measurement. In general, NDIR sensors can suffer errors from infrared absorption of water, as this would effect the reference light measurement. The exact influence of water vapor depends on the specific design with errors ranging from the 100 s to 10 s of ppm over 20–80% relative humidity. For the selected K30 sensor, a recent study has shown that the have uncorrected measurements from this sensor is similar to those from a highly accurate instrument.^44^ When environmental corrections were made, the RMS errors between the K30 sensor and the accurate instrument decreased from about 6 to 2 ppm.^44^ Their work is consistent to prior publication from our laboratory.^37^ Our pervious work found the K30 sensor to be accurate and precise with standard deviation of 1.91 ppm when operating without automatic background calibration. However, when operating with the automatic background calibration turned on (the manufacturer default), the K-30 manufacturer specifications state a maxim error of less than ±30 ppm. This error range is similar to the concentration fluctuation around the average shown in Fig. 8. Given the application of these sensors, no corrections to the ppm valves for temperature, pressure or relative humidity were made. However, such corrections could be made if more accuracy at the atmospheric baseline is desired.

A previous study by Abshire *et al.* at the nearby Department of Energy Atmospheric Radiation Measurement Climate Research Facility (ARM) suggests that low altitude CO_2_ should only increase by roughly 10 ppm compared to the global average reported by the Mauna Loa observatory situated above 3000 m, which is generally stable above 2000 m.^43,45^ The ARM Southern Great Plains site, selected in part for the rural locale, consistently reports diel cycles that peak near 420 ppm, close to the global average.^46^ Local measurement in dense urban centers^47^ have shown excess CO_2_ levels of up to about 80 ppm with much smaller average excesses. Other studies have shown larger excess of up to 100 ppm, with large fluctuation between day and night.^48,49^ The test field location is close to several industry sources, include set of ceramic kilns and a petroleum fractionation tower.

Similar to the daily cycling of temperature, pressure, and other climatological phenomena, relative atmospheric concentration of gases undergoes a diel variation. Determination of these cycles is valuable to climate scientists, biologists, and agricultural engineers for CO_2_.^50–54^ While only a sampling of the available literature is mentioned in the previous citations, a critical analysis of sources shows current technologies are not utilized to detect concentration often, for a long time, and over a large area. These studies usually involve a single sampling instrument operated continuously for a short period, samples taken with large gaps between points, or distributed detection of multiple sites not performed concurrently. The “lightweight” distribution of sensors in the array described here, combined with the relatively short measurement time-scale, lends itself to opportunity for detailed discussion of the diel cycle of reported gas concentrations.

### 4.3 CH_4_ concentration

**Fig. 9:**
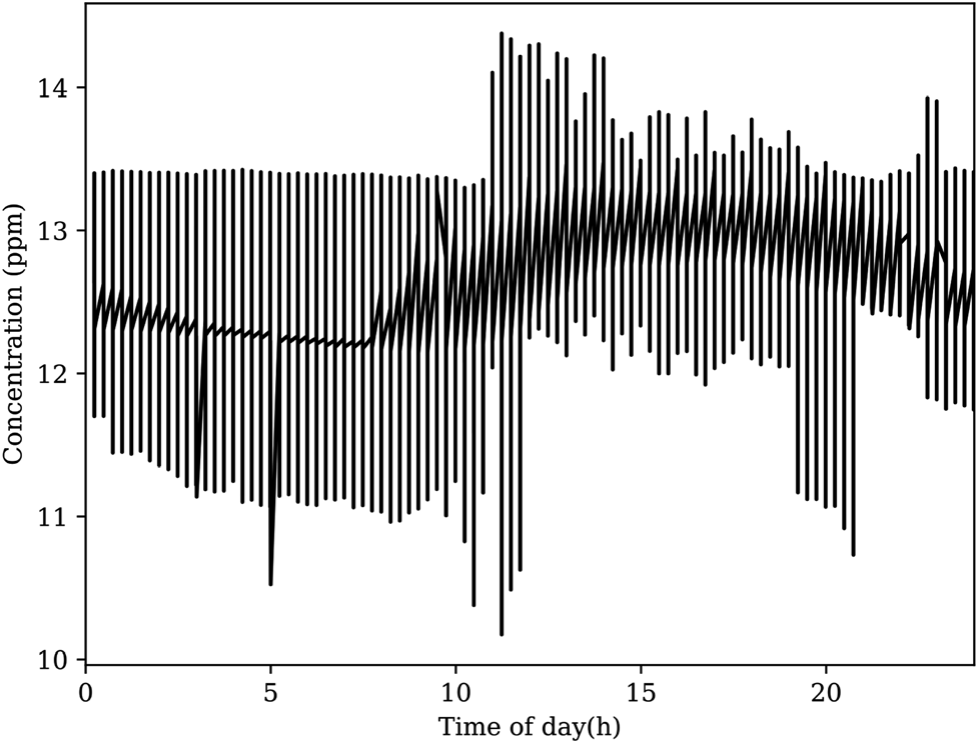
A similar diel cycle appears in the average CH_4_ concentrations reported by the sensor network. The data are noisier than the CO_2_ data due to limitations of the MQ-4 sensors used in the network. The average concentration, even at the lowest reported points, is noticeably greater than the global baseline for CH_4_. However, the reported concentration have significant uncertainty as described in the text.

Similar to our CO_2_ analysis, Fig. 9 shows an averaging of the time of day points for the whole data set as a function of time of day. This figure demonstrates the challenge of constructing low-cost, low-power CH_4_ sensing implementations. Although low cost–cost CO_2_ sensors have good performance,^37^ the same confidence cannot be granted CH_4_ sensors in general. Chemiresistive sensors, which comprise nearly all of the CH_4_ sensors in this class, are notorious for temperature dependence and sensitivity to competing gases, including humidity. In our prior study, we found the MQ-4 sensor was the best performer with regard to precision and accuracy at the low gas concentrations near the atmospheric baseline, and had a limit of detection of 27 ppm.^37^ In addition, the nonlinear calibration of the MQ-4 sensor has signification uncertainty at low concentrations. It is also important to note that for stated application of leak detection, these sensors should be sufficient. The rationale behind the selection was described in Section 2.2.

Similar to the daily cycling reported for CO_2_, daily cycling is also reported for CH_4_.^55–60^ The authors believe that this observation of daily cycling in the CH_4_ data is possible due to the large number of averaged CH_4_ sensors in the network. In terms of the absolute value of CH_4_ concentration reported by the sensors deployed at this site, given the similar values, the elevated CH_4_ background could be real and should be followed up in another study. As OSU is a land-grant school with a strong agricultural program, nearby animal experiments may produce unexpectedly large amounts of GHGs, including CO_2_ and CH_4_. Livestock is a notorious emitter of GHGs.^61–64^ Occupational health and safety and animal husbandry articles often report well-ventilated indoor ranching operations with CO_2_ concentration in thousands of ppm.^65–70^ Despite the accepted estimates of GHG emissions from ranching, there is a dearth of literature reporting ground-level outdoor ambient concentrations based on land use.^71,72^ Satellite remote sensing efforts to date are only able to measure high altitude columns, resulting in distinctions of <5 ppm from heavy urban to light rural land use.^73–75^ Engineering research facilities on the campus, including a concrete testing lab and a chemical engineering pilot plant very near to the deployment site may also contribute to the high gas levels.^76^ Due to Stillwater's relatively rural location in north-central Oklahoma, nearby oil infrastructure may also influence these readings.^72,77,78^ No matter the source, sensor limitations prevent a definite conclusions about the elevated concentrations.

## 5 Conclusions

The constructed devices of the networked array of sensing units provide a viable method for determining the gas concentration above ground. The objective was to develop a sensor system for monitoring leaks at carbon sequestration and EOR sites in remote areas without maintenance and having significantly low cost. In this paper such a system has described and shown to work reliability for a period of several years without maintenance. In all cases, the sensors in the network reliably report data to the master node. The network programming is resilient enough to store redundant copies of the data across nodes of the network. During prolonged periods of low solar flux to charge the cells, units successfully entered a low-frequency sampling regime or “sleep” mode, depending on the level of power depletion. Sensors were additionally shown to be capable of withstanding dangerous weather and seasonal extremes of temperature.

The sensor network distinguishes itself from larger networks such as Mesonet and CNS, filling a niche between those large implementations and small, single-sensor installations. By design choices reflecting an economy of cost and power consumption, a “lightweight” design architecture is achieved. The low power consumption allows for inexpensive power generation from small solar panels, which, in turn, allows for remote deployment of the sensor array from expensive power infrastructure. The tiered mesh networking architecture makes use of the broad GSM cellular coverage in North America, without incurring band saturation, since most of the units in the network only possess smaller radios. This mesh architecture also lends itself well to flexibility, allowing for diverse local geographic distribution to meet project requirements. Collection and storage of data at the server level (Tier 0) provides data access by scientists far-removed from the actual device installation, giving this “lightweight” network capabilities to match or even integrate with the large data repositories discussed above.

Testing the gases in the region just above the surface of the earth is an essential but challenging part of monitoring the environmental impact of carbon storage sites. The sensor network in this paper measure this region in a consistent manner. However, it is clear that improvements need to be in the field of CH_4_ sensing near the atmospheric baseline. New sensors are needed that are resilient, accurate, and low-cost. Notably, the array is capable of detecting minor local variations of gas within an area. This property makes the devices attractive for the identification of micro-seepage within the radius of an injected plume. Consideration in future deployments must be made to account for the sensitivity of the devices responding to unintended sources. The device network described here is a significantly lower cost and implementation barrier than other micro-seepage monitoring strategies.

## Appendix

### Materials and costs

Table 2 shows the estimated component and construction costs for the communication and sensor nodes. The cost is estimated based on the construction of approximately 15 communication and 120 sensor nodes with the final design. The PCB costs include board and the assembly of all components on the board. The enclosures included batteries and solar arrays required for operation. In this work, an additional $20 was spent on a new battery for the sensor nodes. Based on past experience the labor cost needed to construct the node is 50% of the component costs. Shipping costs were not included.

**Table 2:** Component and estimated construction costs of the sensor and communication nodes

		Sens. node	Comm. node
Control board	PCB	$59.02	$117.91
	Parts	$130.72	$16.51
Sensor board	PCB	$19.28	$19.28
	Parts	$65.49	$65.49
Sensors	MQ-4	$4.90	$4.90
	K-30	$85.00	$85.00
	Gascard	—	$1448.00
Air sampling	3D part	$18.18	—
	Pumps	—	$255.21
Cell board	PCB	—	$33.00
	Parts	—	$204.06
Enclosure		$165.56	$1099.96
Construction		$274.00	$1750.00
**Total**		**$548.15**	**$3501.32**

### Device enclosures

**Fig. 10:**
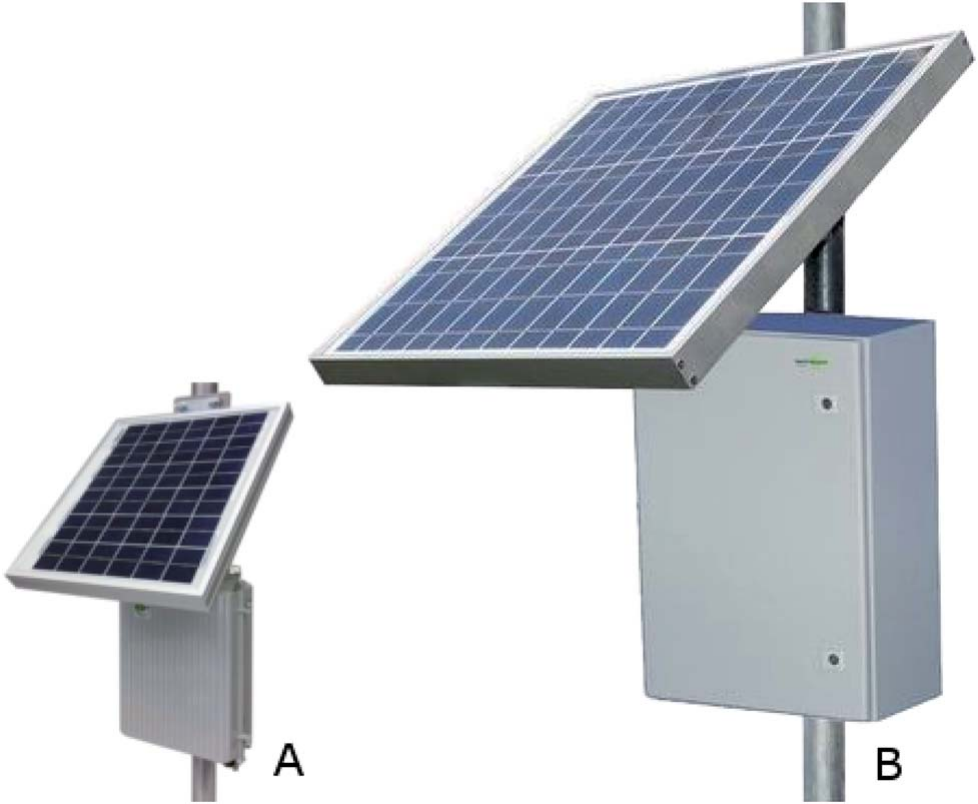
The (A) Remote Pro 2.5 W continuous remote power system and (B) Remote Pro 15 W continuous remote power system. Image adapted from Tycon Power Systems' website.

The Tier 2 devices, the majority of the units in a deployment, were housed in the Remote Pro 2.5 W continuous remote power system die cast enclosure (Fig. 10A) and Tier 1 electronics were housed in the Remote Pro 15 W continuous remote power system steel enclosure (Fig. 10B). The 2.5 W enclosure includes a 12 V battery rated for 9 A h, a charging and distribution circuit, and a 10 W solar panel. The 15 W enclosure includes two 12 V batteries rated for 98 A h, a charging and distribution circuit, and a 60 W solar panel.^79^

### Printed circuit boards

**Fig. 11:**
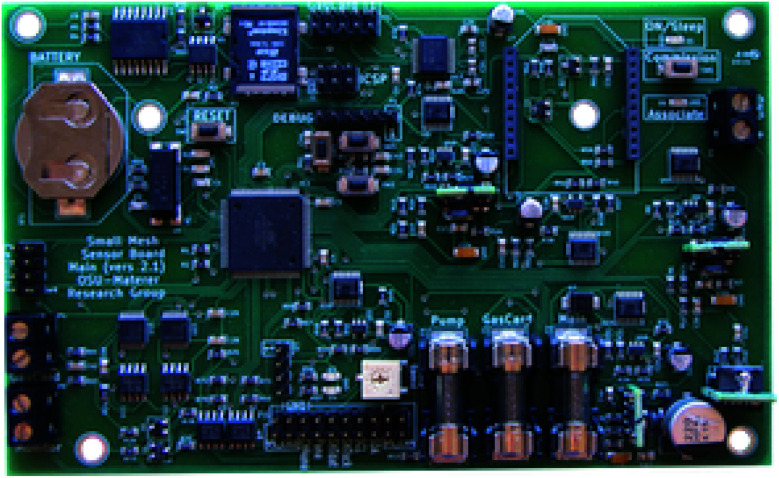
The fully built communication node control board.

**Fig. 12:**
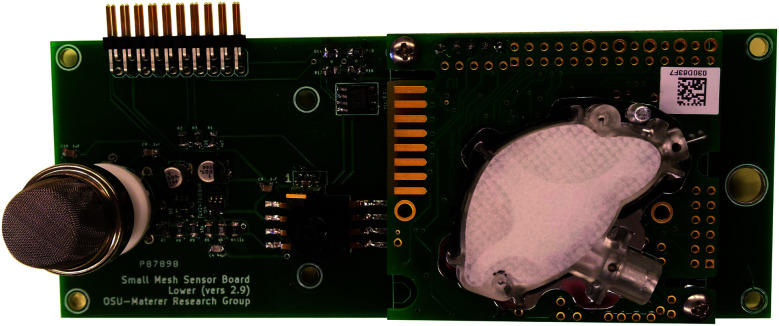
A picture of the sensor board with encapsulating materials removed.

Fig. 11 shows the final control board with all components included. This board is included in all of the high-level communication nodes in the network. Sensor nodes use the same printed circuit board, but do not have electrical components included for unit parts not incorporated into the sensor nodes, such as the Gascard sensor and cellular modem. Each sensing node in the network contains a K-30 CO_2_, temperature and humidity, pressure, and MQ-4 CH_4_ sensors attached to a breakout board, as shown in Fig. 12.

### Passive sampling environmental protection

**Fig. 13:**
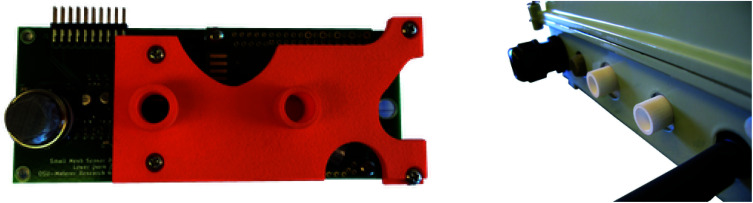
Top and side view of the 3D printed part attached to the standoffs on the sensor board, showing the flush interface with the board and the carbon dioxide sensor.

The sensors are placed in a manner such that they would have contact with the environment for active sampling through holes pre-drilled on the enclosures. These holes are oriented towards the ground to prevent collection of rainwater and moisture. A 3D printed part designed in OpenSCAD^80^ intercedes as a barrier between the board and the metal case (see Fig. 13). By flush mounting of this printed part and sealing exposed areas with epoxy compound, the interior of the enclosure is protected from the elements.

### Stillwater, OK deployment

**Fig. 14:**
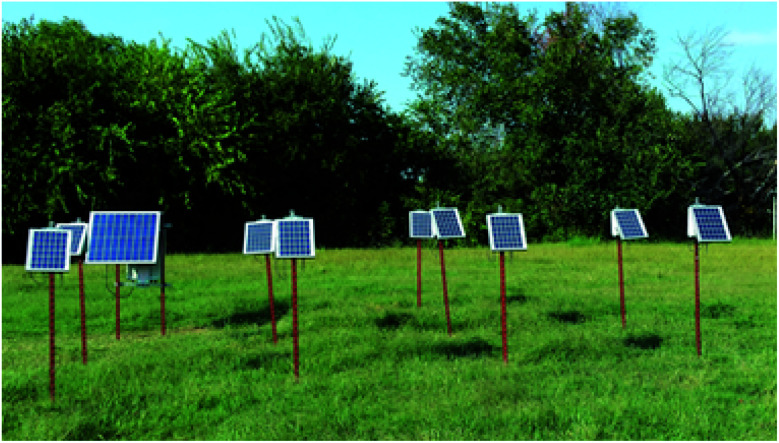
Sensors deployed in a field testing site at Oklahoma State University.

The network of sensors described in this paper, pictured in Fig. 14, was deployed on the north end of the Oklahoma State University campus. Sensors were mounted on fencing T-posts approximately 1 m above the ground. A simple adapter was also developed to allow them to be safely mounted to utility poles using a metal tie strap. The sensors were arranged in a grid, with 3 m spaces between each T-post.

## Conflicts of interest

There are no conflicts to declare.

## Supplementary Material
